# Islets co-engineered with thrombomodulin and CD47 achieve sustained survival in allogeneic recipients without chronic immunosuppression

**DOI:** 10.1172/jci.insight.200686

**Published:** 2026-03-17

**Authors:** Shadab Kazmi, Mohammad Tarique, Darshan Badal, Vahap Ulker, Ali Turan, Kathleen M. Yee-Flores, Abdalmonam Jadou Nejma, Esma S. Yolcu, Haval Shirwan

**Affiliations:** Department of Molecular Microbiology and Immunology, Department of Pediatrics, NextGen Precision Health Institute, Ellis Fischel Cancer Center, University of Missouri, Columbia, Missouri, USA.

**Keywords:** Autoimmunity, Immunology, Islet cells, Tolerance, Transplantation

## Abstract

Allogeneic islet transplantation is an effective treatment for type 1 diabetes, but its clinical use is limited by rejection involving innate and adaptive immune responses, requiring lifelong immunosuppression. We herein engineered islets that transiently display 2 immunomodulators chimeric with streptavidin (SA), thrombomodulin (SA-TM) and CD47 (SA-CD47), for localized modulation of both innate and adaptive immune responses. The engineering process did not impact islet viability, glucose responsiveness, and metabolic activity. Intraportal transplantation into allogeneic recipients achieved sustained survival, with 8 out of 11 grafts surviving 120–330 days without immunosuppression. In contrast, non-engineered islets were acutely rejected (median survival time [MST] = 12 days), while islets engineered with SA-TM showed delayed rejection (MST = 13.5 days) and those with SA-CD47 exhibited prolonged survival (MST = 24 days). Double-engineered islets generated a localized tolerogenic immune environment characterized by low frequencies of inflammatory innate immune cells and increased frequencies of M2 macrophages, myeloid-derived suppressor cells, and CD4^+^FoxP3^+^ T regulatory cells. The transcriptomic analysis showed downregulation of proinflammatory and upregulation of immune regulatory pathways. Our results demonstrate that transient co-display of immunomodulatory molecules on the islet surface is a versatile platform with significant translational potential for islet transplantation.

## Abstract 2

Engineering transplanted islets with thrombomodulin and CD47 protects them from immune attack, enabling long-term survival in diabetic recipients without the need for chronic immunosuppressive drugs.

## Graphical abstract

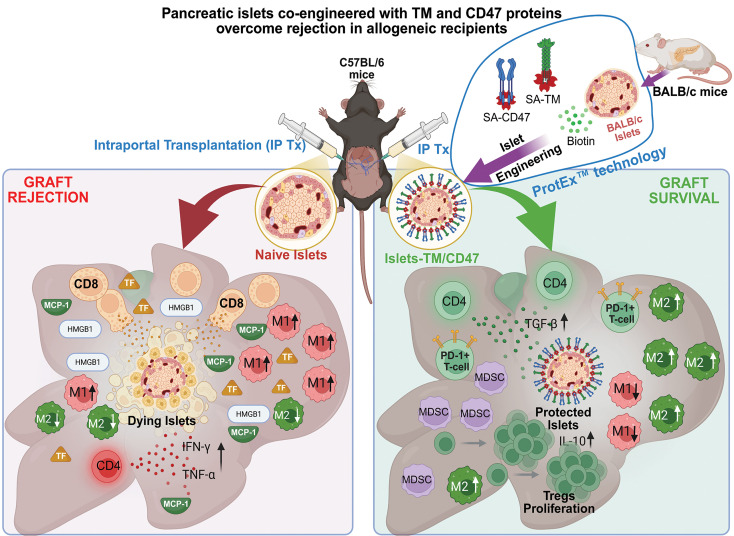

## Introduction

Type 1 diabetes (T1D) represents a complex autoimmune disorder characterized by selective destruction of insulin-producing β cells, resulting in lifelong insulin dependence without a cure. Islet replacement has emerged as an effective therapy for T1D (1–3). However, immune rejection remains the primary hurdle for its broad clinical application, which necessitates lifelong immunosuppression with significant adverse effects. Islet graft destruction is mediated by 2 distinct but overlapping immune responses: innate proinflammatory, defined as instant blood-mediated inflammatory reaction (IBMIR), and adaptive, primarily mediated by T cells (4, 5). IBMIR is responsible for the destruction of a significant islet graft mass, ranging from 50% to 70%, immediately after intraportal transplantation (6, 7). As such, islets from multiple donors are required to achieve euglycemia in allogeneic recipients (8, 9). Islets that survive innate immunity are subsequently subjected to rejection by adaptive immune responses, which are managed through lifelong chronic immunosuppression (10, 11). An immunomodulatory approach that effectively modulates both innate and adaptive immune responses has the potential to obviate the need for both islets from multiple donors and lifelong immunosuppression.

Thrombomodulin (TM), an endothelial surface glycoprotein, regulates hemostasis and inflammation by forming complexes with thrombin to activate protein C that degrades clotting factors Va and VIIIa, which are culprits of early islet mass destruction (12–14). TM also has additional antiinflammatory properties, including inhibition of leukocyte adhesion and modulation of proinflammatory cytokines (15). CD47 is a key modulator of both innate and adaptive immune responses by interacting with SIRP-α on phagocytes, including dendritic cells (DCs), which are essential for generating adaptive immune responses. Signaling through SIRP-α serves as a “do not eat me” signal to prevent phagocytosis by macrophages and DCs. SIRP-α signaling has also recently been shown to inhibit NK cells (16, 17). Overexpression of CD47 has been shown to significantly suppress immune responses by reducing IFN-γ, leading to the inhibition of donor-specific IgM antibodies and cytotoxic activity of both T cells and NK cells (18, 19). Thus, targeted simultaneous modulation of both TM and CD47 pathways has the potential to modulate both innate and adaptive alloreactive immune responses with the potential of achieving sustained islet graft survival.

Islet grafts contribute to their own demise by expressing tissue factor (TF), various danger-associated molecular patterns, and proinflammatory cytokines and chemokines that collectively lead to the activation of coagulation, complement, and innate immune pathways (20–22). Modulating islets ex vivo prior to transplantation offers an opportunity to mitigate destructive pathways and guide the immune system toward protection. Our group pioneered the concept of transient display of recombinant immune modulators on the islet surface as a practical alternative to gene therapy for localized immunomodulation (23). The approach involves designing expression constructs in which coding sequences for extracellular functional domains of immune ligands are fused to either the 5′ or 3′ end of a modified form of streptavidin (SA) coding sequences. The constructs are expressed in insect cells, and recombinant chimeric immune ligands are attached to the biological surfaces that have been modified with biotin, taking advantage of the high-affinity interaction (*K*_d_ ≈ 10^–14^ M) between biotin and SA (24). This technology ensures stable surface display while maintaining the function of immune ligands with demonstrated immunomodulatory efficacy in various settings (13, 23, 25–30).

We previously demonstrated that islet grafts engineered with either SA-CD47 or SA-TM had improved engraftment in an intraportal minimal mass model of syngeneic transplantation (7, 18). Co-engineering with both molecules resulted in better engraftment efficacy compared with single engineering (13). Engraftment efficacy correlated with a significant decrease in innate effector cells and soluble mediators of IBMIR (7, 13). Given the function of TM and CD47 in modulating innate and adaptive immune responses (31–36), we herein investigated the efficacy of co-engineering islets with both molecules in preventing rejection in an allogeneic mouse model of intraportal transplantation without chronic immunosuppression. Our results demonstrate that islets can be effectively co-engineered with TM and CD47 molecules without significantly affecting their viability and metabolic functions. Engineered islet grafts achieved sustained survival in allogeneic hosts by downregulating destructive innate and adaptive immune responses while upregulating protective regulatory immune responses.

## Results

### Islets co-engineered with SA-TM and SA-CD47 maintain viability and function.

**Figure 1:**
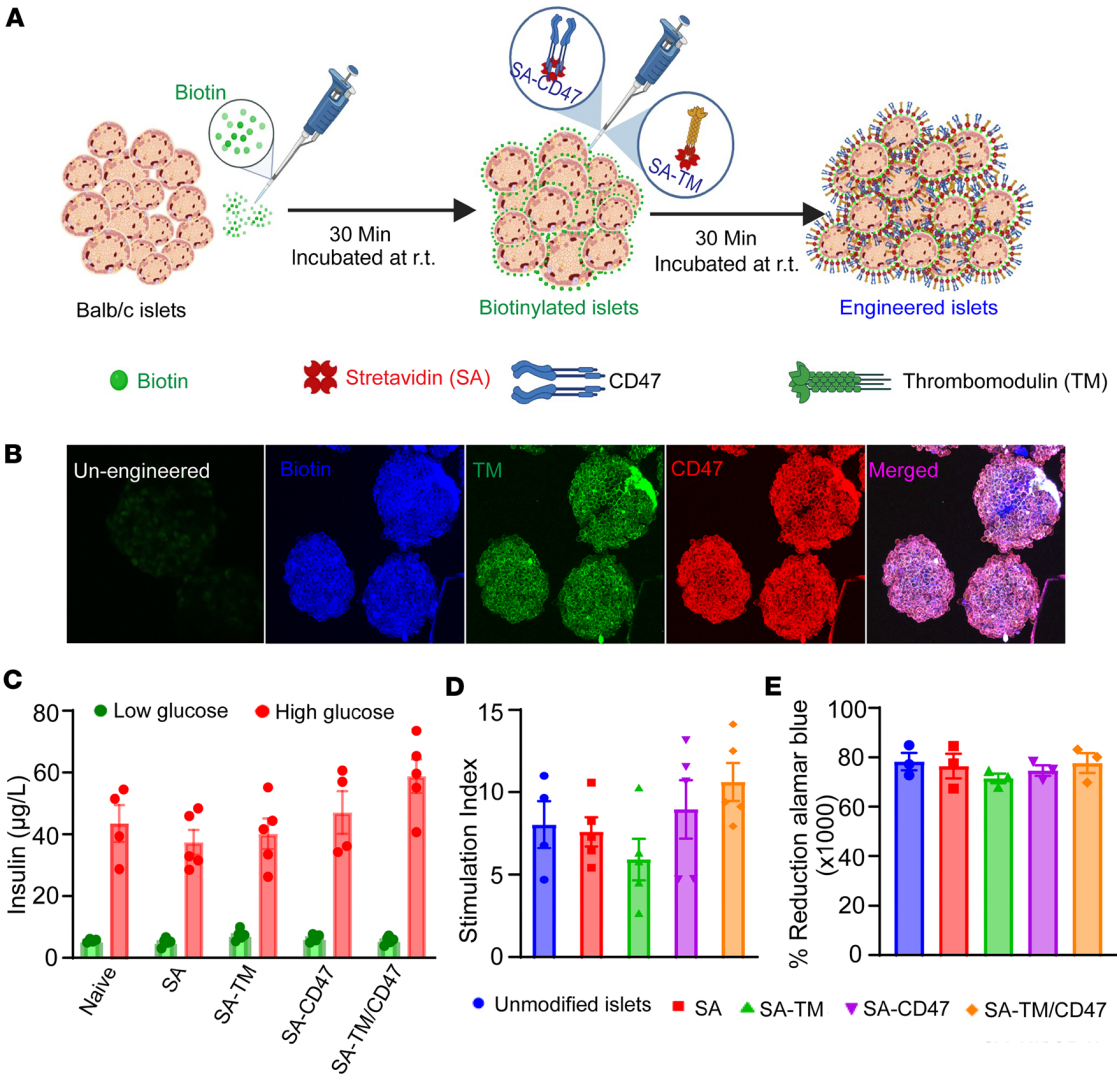
Engineering islets with SA-TM and SA-CD47 proteins does not affect their viability or function. (**A**) Schematic illustration showing stepwise engineering of pancreatic islets. BALB/c pancreatic islets were surface modified with biotin followed by incubation with SA-chimeric proteins. (**B**) Representative images of double SA-TM and SA-CD47 co-engineered islets. Islets were biotinylated (15 μM) followed by co-engineering with 6.2 μg SA-TM and 0.8 μg SA-CD47 per 1000 islets. Confocal microscopy images of double-engineered islets using anti-SA–Texas red (blue), anti–human TM–FITC (green), anti-CD47–APC (red), and merged images and un-engineered islets used as negative control. (**C**) Glucose-stimulated insulin secretion conducted on islets without engineering and those engineered with single or both proteins. Islets were cultured in low (3.5 mM) and high (16.5 mM) glucose concentrations followed by the quantification of secreted insulin using ELISA (*n* = 4–5). (**D**) Stimulation index (mean secreted insulin in high glucose/mean secreted insulin in low glucose) of the data shown in **C**. (**E**) Cell viability and functional activity of engineered islets assessed using an Alamar blue cell viability assay (*n* = 3). Data are presented as mean ± SD with no statistically significant differences as assessed using 1-way ANOVA with Tukey’s post hoc test.

In previous studies, we reported successful co-display of SA-TM and SA-CD47 proteins on the surface of islets from C57BL/6 mice without compromising their function and engraftment in a minimal mass intraportal syngeneic transplantation model (13). Given potential strain-specific variations and also to demonstrate the broader applicability of our engineering approach, BALB/c islets were co-engineered using SA-TM and SA-CD47 proteins (SA-TM/CD47) at the previously optimized doses of 3.2 μg and 0.4 μg per 500 islets, respectively (Figure 1A) (13). Confocal microscopic analysis using fluorescein-labeled antibodies against each molecule demonstrated successful co-engineering of islets (Figure 1B). We next investigated whether co-engineering with both proteins has a negative impact on islet viability and function. Co-engineered islets demonstrated a comparable glucose-stimulated insulin secretion response to that of unmodified and single-agent engineered islets (Figure 1, C and D). Islets engineered with individual proteins or in combination exhibited metabolic function at levels comparable to those of unmodified islets (Figure 1E). These results show that co-engineering islets with SA-chimeric immune ligands are effective across different mouse strains and do not negatively affect islet viability or function.

### Islets engineered with SA-TM and SA-CD47 demonstrate sustained long-term survival in allogeneic recipients.

**Figure 2:**
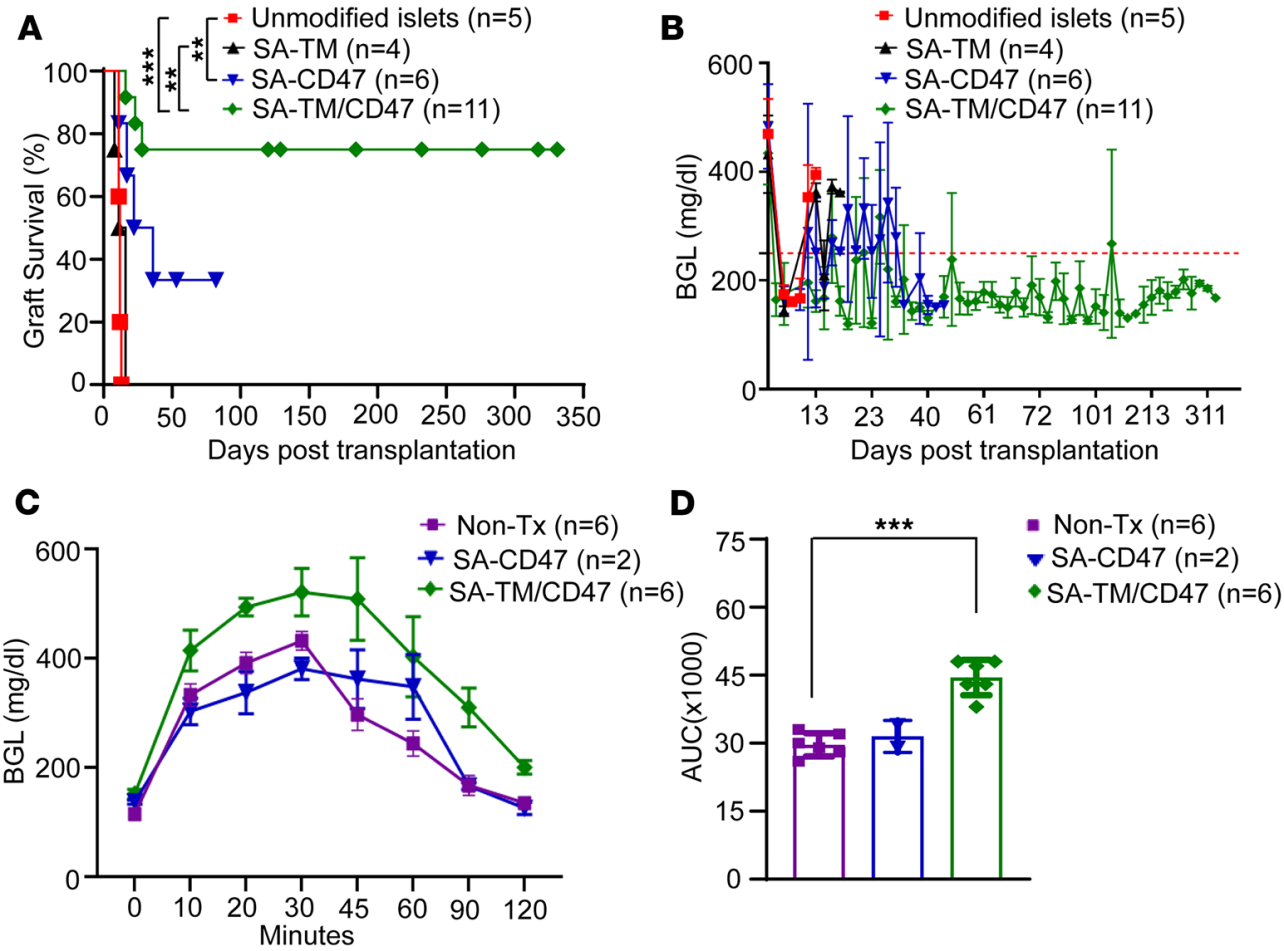
Islets engineered to transiently co-display SA-TM and SA-CD47 proteins on their surface achieve long-term survival in diabetic allogeneic recipients. BALB/c islets were engineered with individual or a combination of SA-TM and SA-CD47 proteins, and 700 IEQ were transplanted intraportally into streptozotocin-induced diabetic C57BL/6 recipients. Naive (unmodified islets) were used as controls. Recipients were monitored for blood glucose levels, and those exceeding 250 mg/dL on 2 consecutive days were considered rejected. (**A**) Graft survival. **P < 0.01; ***P < 0.001 by log-rank (Mantel-Cox) test. (**B**) Non-fasting blood glucose levels (BGL) of the indicated graft recipients. (**C**) IPGTT was performed on long-term recipients of islets engineered with SA-CD47 (*n* = 2; 52 and 108 days after transplantation) and SA-CD47/TM (*n* = 1, 126 days; *n* = 5, > 226 days after transplantation), with naive mice without transplant serving as controls. (**D**) The area under the curve (AUC) was calculated for the data presented in **C**. Tx, transplantation. Data are presented as mean ± SD. ***P < 0.001 by unpaired, 2-tailed Student’s *t* test.

We next tested the efficacy of engineered islets for long-term survival following intraportal transplantation in allogeneic hosts. BALB/c islets without engineering or engineered with single proteins or SA-TM/CD47 were transplanted intraportally into streptozotocin-induced (STZ-induced) diabetic C57BL/6 allogeneic recipients (37). SA-TM/CD47–engineered BALB/c islets established euglycemia and long-term survival, with 8 out of 11 grafts showing no rejection during an observation period ranging from 120 to 330 days (Figure 2, A and B). In marked contrast, islet grafts engineered with SA-TM alone normalized blood glucose levels after transplantation; however, all were rejected acutely (median survival time [MST] = 13.5) at a rate similar to non-engineered controls (MST = 12). SA-CD47–engineered islets had significant prolongation (MST = 24 days), with 2 out of 6 grafts surviving for an observation period of 53 and 82 days when they were euthanized for mechanistic studies. Intraperitoneal glucose tolerance test (IPGTT) results at the endpoint confirmed the fitness of SA-TM/CD47–engineered islet grafts in clearing blood glucose levels (Figure 2C). However, the area under the curve analysis showed less effective blood glucose clearance as compared with naive or long-term-surviving SA-CD47–engineered islet graft recipients (Figure 2D). This difference may be attributable to the age of grafts, as double-engineered islet grafts were assessed on days 120–330 versus 53–82 for SA-CD47–engineered islet grafts. These results demonstrate that co-engineering islets with SA-TM and SA-CD47 is an effective approach to modulating alloreactive immune responses to achieve sustained long-term graft survival.

### Islets co-engineered with SA-TM and SA-CD47 polarize immune responses toward graft-protective regulatory responses.

**Figure 3:**
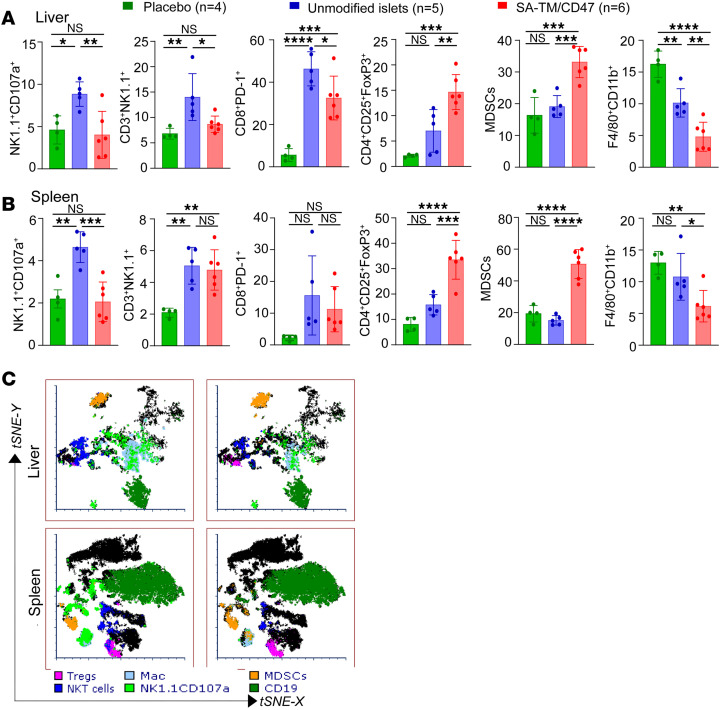
Deep immune monitoring of SA-TM/CD47–engineered islet recipients 3 days after transplantation shows immune polarization toward graft-protective responses. Diabetic C57BL/6 mice were transplanted intraportally with 700 IEQ per recipient allogenic naive islets or those engineered with a combination of SA-TM and SA-CD47 proteins. Placebo controls included non-transplanted, non-diabetic C57BL/6 mice. Islet-graft-harboring liver and spleen were harvested on day 3 after transplantation. Immune cells were stained with fluorescently labeled Abs to the indicated cell surface markers and analyzed using flow cytometry. (**A** and **B**) Frequency of the indicated immune cell populations plotted as percentages for the liver and spleen, respectively. (**C**) t-SNE plots of immune cells harvested from the liver and spleen. Data represent the percentage of immune cells per tissue shown as mean ± SD from *n* = 5–6 graft recipients and *n* = 4 placebo controls. *P < 0.05; **P < 0.01; ***P < 0.001; ****P < 0.0001 by 1-way ANOVA with Tukey’s post hoc test. NS, not significant.

We next conducted a time-course deep immunophenotyping study to elucidate the mechanistic underpinnings of SA-TM/CD47–mediated sustained graft survival. Splenocytes and liver-infiltrating immune cells harvested on days 3 and 12 after transplantation were stained with fluorochrome-labeled antibodies to identify various immune cell subtypes using flow cytometry. Age-matched, non-transplanted, non-diabetic C57BL/6 mice were included as placebo controls to establish baseline immune cell populations for study on day 3. On day 3, there was a significant reduction in the percentage and absolute cell numbers of NK cells expressing the degranulation marker CD107a (*P* < 0.01) and NKT cells (*P* < 0.05) in the liver and spleen (NKT percentage was not significant) of recipients of double-engineered islets as compared with the control group (Figure 3, A and B, and Supplemental Figure 2, A and B; supplemental material available online with this article; https://doi.org/10.1172/jci.insight.200686DS1). Notably, placebo control mice exhibited NK and NKT cell immunophenotypic profiles comparable to that observed in recipients of SA-TM/CD47–engineered islets, suggesting a normalization toward baseline immune cell distributions. There was also a significant reduction in the percentage and absolute cell number of CD11b^+^F4/80^+^ macrophages in the liver and spleen of recipients of double-engineered islets as compared with controls (Figure 3, A–C, and Supplemental Figure 2, A and B). In contrast, placebo control mice showed significantly elevated macrophage levels in the liver, whereas no significant difference was observed in the spleen. In marked contrast, we observed increased frequency and absolute cell numbers for myeloid-derived suppressor cells (MDSCs, CD45^+^CD11b^+^Ly6G^+^Ly6C^+^) and FoxP3^+^ Treg cells in both the spleen and liver of double-engineered islets as compared with controls as well as placebo (Figure 3, A and C, and Supplemental Figure 2, A and B). CD8^+^PD-1^+^ T cells showed a significant decrease (*P* < 0.05) in frequency in the liver (Figure 3A), but not spleen (Figure 3B), and there were no changes in absolute cell numbers in either organ (Supplemental Figure 2, A and B). Notably, CD8^+^PD-1^+^ T cells were significantly increased in the liver of recipients of unmodified islets as well as SA-TM/CD47–engineered islets, whereas no significant changes were observed in the spleen (Figure 3, A and B).

**Figure 4:**
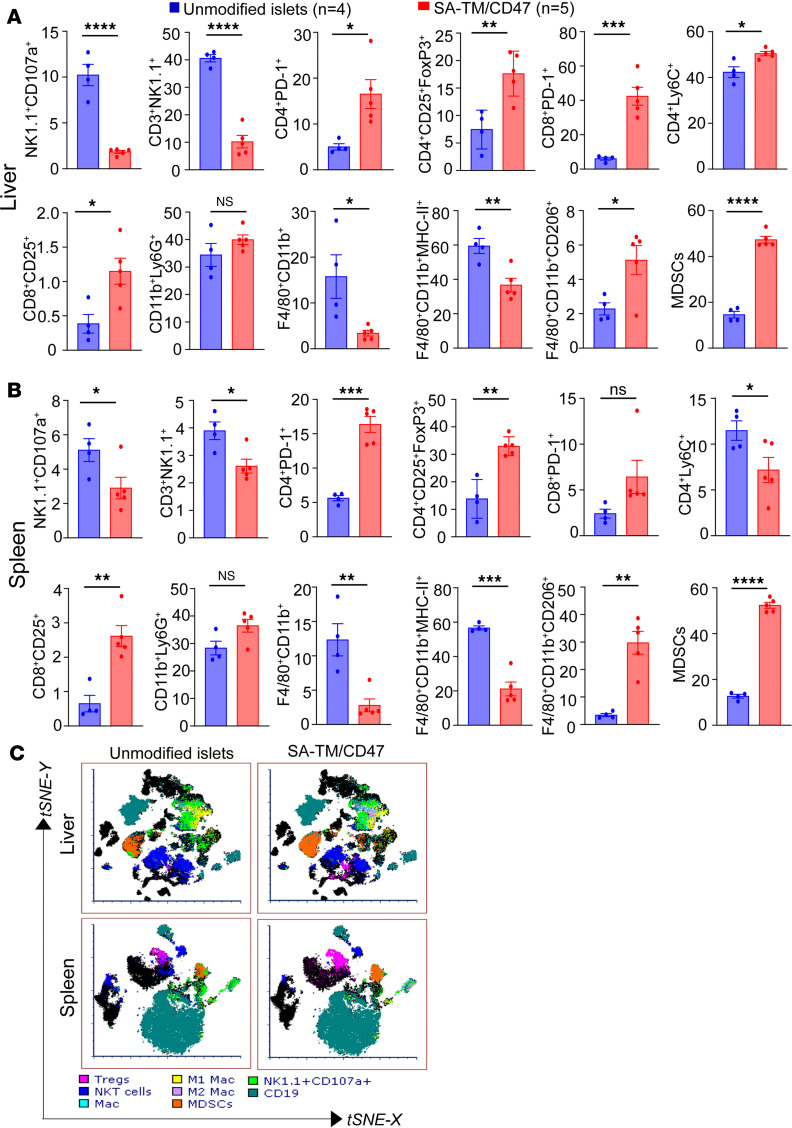
Deep immune monitoring of SA-TM/CD47–engineered islet recipients 12 days after transplantation shows an elevated level of immune polarization toward graft-protective responses. Experimental procedures and data analysis are as in Figure 3, except that tissues were harvested 12 days after transplantation. (**A** and **B**) Percentage of the immune cell population in the liver and spleen, respectively. (**C**) t-SNE (FCS express) plots of immune cell populations in the liver and spleen. Data represent the percentage of immune cells per tissue shown as mean ± SD from *n* = 4–5 graft recipients. *P < 0.05, **P < 0.01, ***P < 0.001, ****P < 0.0001 by 2-tailed, unpaired Student’s *t* test. NS, not significant.

**Figure 5:**
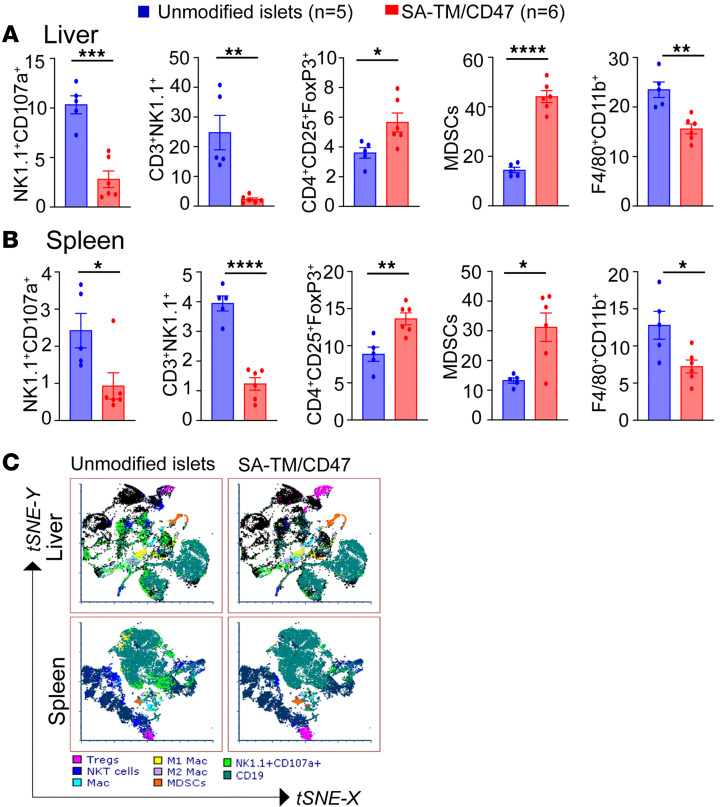
Long-term recipients of SA-TM/CD47–engineered islets have high frequencies of Tregs and MDSCs in the graft site and spleen. Immune cells were harvested from long-term graft recipients (*n* = 2, 130 days; *n* = 1, 186 days; *n* = 1, >223 days; *n* = 2, 277 days after transplantation) and analyzed for various immune cell types, as detailed in Figure 3. (**A** and **B**). Cells from recipients of unmodified islet grafts at rejection served as control. Percentage of immune cell population in the liver and spleen, respectively. (**C**) t-SNE plots of immune cell populations in the liver and spleen. Data represent the percentage of immune cells per tissue shown as mean ± SD from *n* = 5–6 graft recipients. *P < 0.05, **P < 0.01, ***P < 0.001, ****P < 0.0001 by 2-tailed, unpaired Student’s *t* test.

Flow cytometric analysis of graft-infiltrating immune cells in the liver on day 12 after transplantation showed a pattern like that on day 3, with some pronounced differences. The frequency (Figure 4, A–C) and absolute numbers (Supplemental Figure 3, A and B) of NK1.1^+^CD107^+^ NK and NKT cells were significantly reduced in both the liver and spleen of the recipients transplanted with double-engineered islets compared with controls. There was also a marked reduction in the frequency of M1 proinflammatory (F4/80^+^CD11b^+^MHC II^+^) and an increase in M2 (F4/80^+^CD11b^+^CD206^+^) regulatory macrophages and MDSCs in the liver of recipients of double-engineered islets (Figure 4, A–C). Spleen showed a similar pattern, except for the frequencies of CD8^+^PD-1^+^ T cells that were not significantly different between the 2 groups. The changes in the frequencies of immune cell types are also reflected in the absolute cell numbers for a given population for both the liver and spleen (Supplemental Figure 3). Immune phenotyping studies on long-term (120–330 days) surviving mice demonstrated significantly decreased frequencies of NK1.1^+^CD107a^+^ NK and NKT cells and increased frequencies of regulatory populations, including FoxP3^+^ Tregs and MDSCs (CD45^+^CD11b^+^Ly6G^+^Ly6C^+^) in both tissues (Figure 5, A–C).

### SA-TM/CD47–engineered islets enhance the expression of graft-protective regulatory genes while inhibiting those associated with rejection.

**Figure 6:**
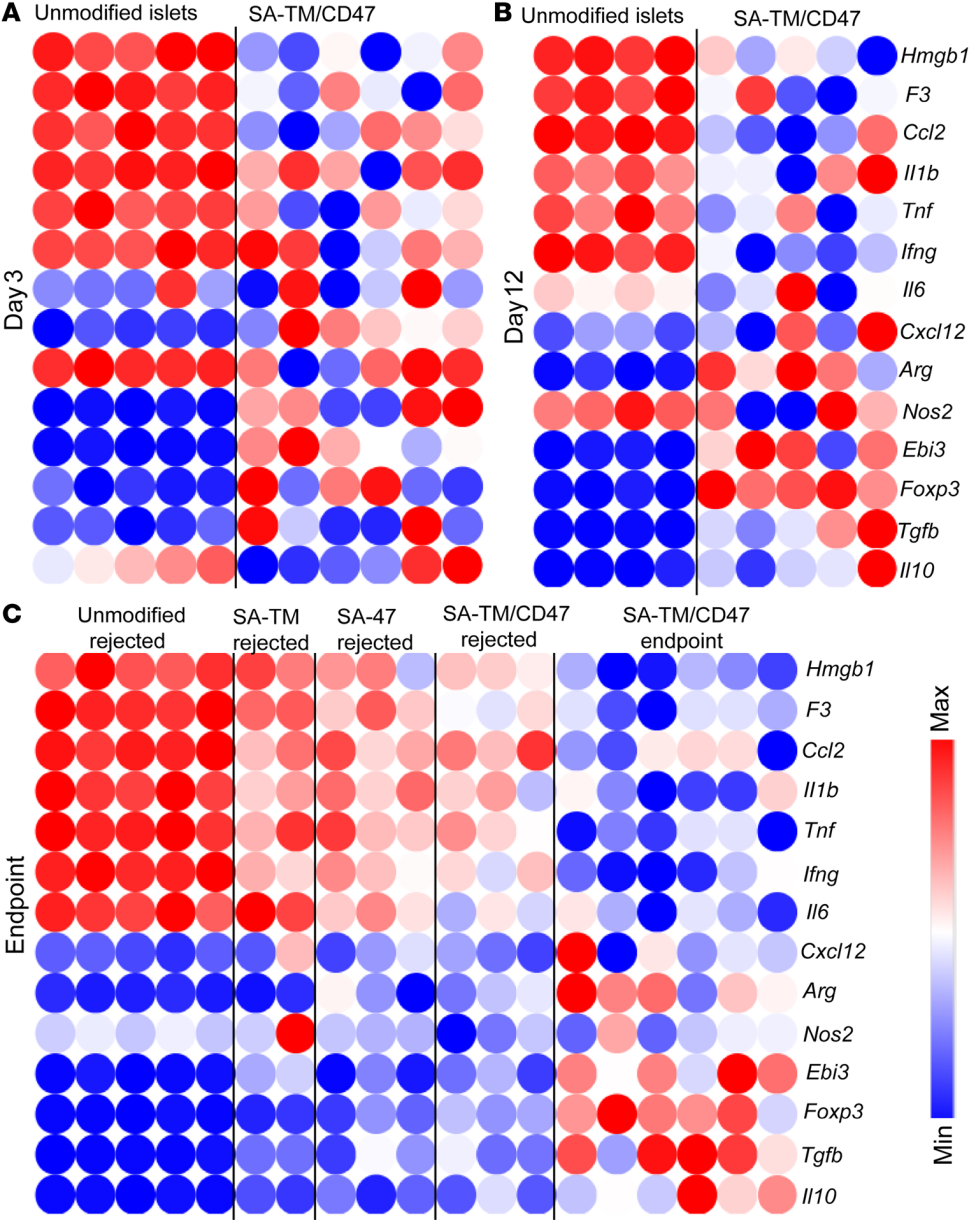
Real-time quantitative PCR analysis of target genes in the SA-T/CD47–engineered islet graft site at various time points after transplantation reveals immune polarization toward a protective response. Total RNA was extracted from the liver of recipients of islet grafts engineered with individual molecules or in combination on day 3, 12, and endpoint (rejection or long-term survival) and analyzed for the expression of pro- and antiinflammatory genes using TaqMan qPCR. Total RNA extracted from the liver of recipients transplanted with unmodified islets served as a control. Heatmaps of genes from day 3 (**A**), day 12 (**B**), and at rejection or end-point (*n* = 2, 130 days; *n* = 1, 186 days; *n* = 1, >223 days; *n* = 2, 277 days after transplantation) (**C**). Expression levels were normalized to housekeeping genes and compared between and among the groups. Morpheus (https://software.broadinstitute.org/morpheus/) was used to generate the heatmaps.

Quantitative RT-PCR was performed on total RNA extracted from islet-graft-harboring liver to assess the expression of anti- and proinflammatory genes on days 3 and 12 after transplantation, at rejection, or at the 120- to 330-day experimental endpoint for a more in-depth understanding of the mechanistic basis of the immunomodulatory efficacy of double-engineered islet grafts. The gene expression profile corroborated the findings from immune cell phenotyping studies. Proinflammatory genes, including *Hmgb1*, *F3* (tissue factor), *Ccl2*, *Il1b*, *Tnfa*, and *Il6* were markedly downregulated in recipients of SA-TM/CD47–engineered islet grafts as compared with those in the recipients of unmodified islet grafts (Figure 6, A and B). In marked contrast, there was increased expression of immunoregulatory genes, including *Arg*, *Ebi3*, *Foxp3*, *Tgfb*, and *Il10*, indicating a transcriptional shift toward a tolerogenic immune phenotype (Figure 6, A and B). This pattern of gene expression remained consistent across all time points analyzed, including long-term grafts (Figure 6C). Recipients of islet grafts engineered with individual or both molecules showed similar gene expression patterns in the liver at rejection compared with the recipients of unmodified grafts (Figure 6C). Notably, EBi3, a key subunit of IL-27, was consistently upregulated and may contribute to the expansion and stability of FoxP3^+^ Treg cells. Elevated expression of *Arg*, *ll10*, and *Tgfb* further supports the establishment of a robust antiinflammatory and immunoregulatory transcriptional network. Collectively, these data demonstrate that immunomodulation with SA-TM and SA-CD47 is effective in driving the expression of genes involved in immune tolerance.

### Long-term graft survivors retain systemic response to donor alloantigen.

**Figure 7:**
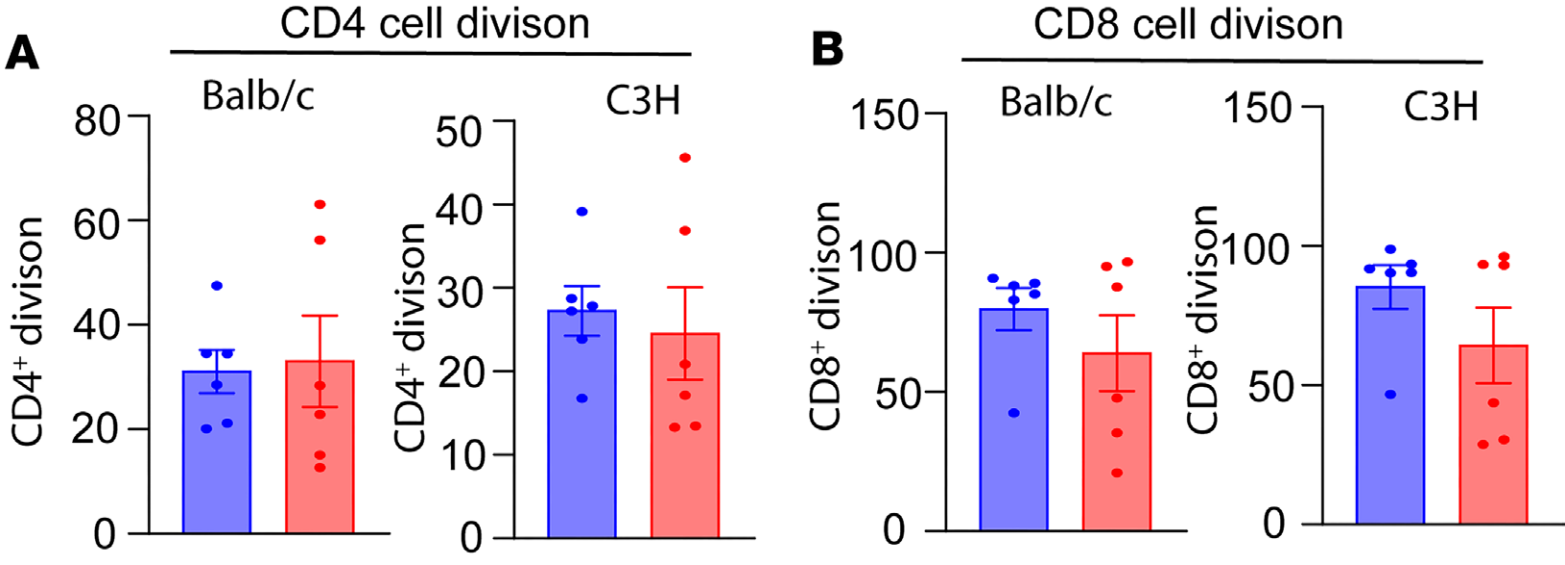
T cells of long-term islet graft survivors generate a normal response to donor antigens. Splenocytes from naive and long-term survivors were used as responders against irradiated donor-matched BALB/c or C3H third-party splenocytes in a standard CFSE-based proliferation assay. Proliferation of CD4^+^ (**A**) and CD8^+^ (**B**) T cells was assessed using Abs against CD3, CD4, and CD8 in flow cytometry and plotted as the percentage division for each cell population. Data presented as mean ± SD. Statistical significance was determined using a 2-tailed, unpaired Student’s *t* test.

Since SA-TM and SA-CD47 were administered locally with the islet graft, this approach is expected to preserve systemic immune responsiveness. This is consistent with our previous studies using allogeneic islets engineered with SA-FasL and SA-PD-L1 (25, 27). To assess this prediction, a CFSE-based ex vivo mixed lymphocyte reaction assay was conducted using splenocytes from naive and long-term recipients as responders to irradiated donor BALB/c or C3H third-party stimulators. CD4^+^ T cells from long-term graft recipients showed a proliferation rate comparable to that of naive mice when stimulated with donor or third-party splenocytes (Figure 7A). CD8^+^ T cells from long-term recipients also generated a proliferative response to donor antigens. Although the response was reduced as compared with cells from naive animals, the diminished response also occurred with the third-party antigens (Figure 7B). The similar proliferation rates observed in both splenic CD4^+^ and CD8^+^ T cells, compared with naive controls, indicate that immunomodulation with SA-TM/CD47–engineered islet grafts is localized to the graft site and does not compromise systemic immune responses.

## Discussion

Intraportal pancreatic islet transplantation is a clinically practiced treatment for individuals with unstable T1D. Islets introduced into the liver trigger two distinct but overlapping immune rejection responses, innate and adaptive. We herein engineered islets to transiently display on their surface 2 immunomodulatory molecules, SA-TM and SA-CD47, as a strategy to regulate both innate and adaptive immune responses in favor of graft survival. Intraportal transplantation of islets engineered with SA-TM and SA-CD47 resulted in long-term survival by inhibiting proinflammatory destructive immune responses while amplifying graft-protective regulatory responses. There was a significant reduction in proinflammatory immune cells, including NK cells, NKT cells, and inflammatory macrophages, while increasing the frequency and absolute numbers of immunosuppressive MDSCs, Tregs, and M2 macrophages. This dual-engineering strategy suppresses graft-destructive innate and adaptive immune responses while inducing graft-protective regulatory responses, resulting in long-lasting graft survival without a need for systemic immunosuppression.

Islets were successfully engineered using our ProtEx platform technology, which involves biotinylation of islets followed by decoration with SA-TM and SA-CD47 under physiological conditions, exploiting the high-affinity interaction between biotin and SA (24). Engineering with both proteins did not compromise islet viability or metabolic functions, consistent with our previous studies using various immunomodulatory ligands (7, 13, 18, 25–27, 29, 38). Islets engineered with both molecules were effective in establishing euglycemia in over 72.7% of diabetic allogeneic recipients long-term. Using SA-TM for single engineering did not increase graft survival, whereas islets engineered with SA-CD47 demonstrated a significant prolongation of survival compared with the unmodified control grafts, with over 33.3% surviving long-term. We speculate this may be due to shorter cell surface persistence of SA-TM (*t*_1/2_ ≈ 6 hours) as compared with SA-CD47 (*t*_1/2_ ≈ 48 hours), as we previously published (7, 18). These findings are consistent with the role of TM in primarily regulating the coagulation pathway by activating protein C, while CD47 prevents phagocyte-mediated islet damage by interaction with SIRP-α. Transgenic expression of CD47 in islet grafts has been shown to mitigate rejection both in allogeneic and xenogeneic recipients (39, 40). Additionally, CD47 promotes immune tolerance by shifting adaptive immune responses toward regulatory mechanisms, which include the inhibition of DC function and the generation of both Tregs and MDSCs (34–36, 41). The observed synergistic effect of SA-TM and SA-CD47 is consistent with the function of TM primarily regulating coagulation, a trigger of IBMIR that causes significant islet mass loss immediately after transplantation, and CD47 inhibiting adaptive effector responses while inducing regulatory responses.

Long-term graft survival was associated with a significant reduction in the frequency and absolute numbers of NK1.1^+^CD107a^+^ cells in the liver, the graft site, and the spleen, as assessed on days 3 and 12 after transplantation. CD107a is a marker of degranulation, which reflects NK cell cytotoxic activity (42). The reduction in CD107a^+^ cells is consistent with CD47 serving as an immune checkpoint to inhibit NK cell activation and cytotoxicity through engagement with SIRP-α (33, 43). Similarly, NKT cells also showed a significant reduction in both frequency and absolute numbers in the liver and spleen. NKT cells have been shown to play a critical role in the rejection of allogeneic islet grafts by engaging in cross-communication with hepatic monocytes that respond to HMGB1 stimulation (20). The observed synergistic efficacy of SA-TM and SA-CD47 in sustaining long-term islet graft survival is consistent with TM blocking the function of HMGB1 released from the graft to stimulate monocytes and macrophages through Toll-like receptors (44–46) and CD47 serving as an immune checkpoint inhibitor for both macrophages and NK and NKT cells (47).

We also observed a significant reduction in the frequency and absolute numbers of inflammatory macrophages (CD11b^+^F4/80^+^MHC II^+^) in both the liver and spleen in long-term graft survival, as well as on day 3 and day 12 after transplantation. M1 macrophages produce proinflammatory cytokines such as IL-1β, TNF-α, and IL-6, which contribute to tissue inflammation and adaptive immune activation (48). The decrease in proinflammatory cytokines IL-1β, IL-6, and TNF-α in our study is consistent with the function of TM in reducing endothelial activation and leukocyte recruitment, thereby limiting macrophage infiltration into the graft microenvironment (7, 49). TM has been shown to reduce leukocyte adhesion and migration by interfering with P-selectin and ICAM-1 signaling (50). The reduction in inflammatory macrophages was accompanied by a significant increase in the frequency and absolute numbers of regulatory macrophages (CD11b^+^F4/80^+^CD206^+^) in the liver. M2 macrophages release antiinflammatory cytokines such as IL-10 and TGF-β, which were upregulated in the graft site in our study and promote tissue repair and immunosuppression. The increased frequency of M2 macrophages in the graft site and spleen of recipients transplanted with SA-TM/CD47–engineered islet grafts implicates TM, which induces M2 polarization by modulating endothelial cell signaling and reducing the local inflammatory environment (51).

The increase in tolerogenic immune cells extended beyond macrophages. A marked increase in the frequency and absolute numbers of MDSCs was observed in both the liver and spleen of recipients transplanted with double-engineered islets. MDSCs are known to suppress T cell activation and proliferation through the PD-1/PD-L1 signaling axis and metabolic regulation involving arginase-1 (Arg1) (52). PD-L1 engagement with PD-1 on T cells dampens T cell receptor signaling, leading to reduced cytokine production and impaired T cell proliferation. Arg1 depletes L-arginine, which is required for T cell activation and function, thereby reinforcing the immunosuppressive activity of MDSCs (53). The increased presence of MDSCs suggests that CD47 overexpression enhanced MDSC recruitment and stabilization within the graft microenvironment. This finding is consistent with previous studies demonstrating that CD47 promotes MDSC expansion and activity in tumor models and transplant settings (34, 39, 54, 55). MDSCs also crosstalk with Tregs by inducing them through the production of IL-10 and TGF-β, which in turn affect the proliferation and maintenance of MDSCs (56, 57). We observed a significant increase in the frequency and absolute numbers of Tregs in both the liver and spleen. The frequency and absolute number of CD8^+^ T cells expressing the exhaustion marker PD-1 in the graft site, but not in the spleen, were significantly higher in recipients transplanted with double-engineered islets, demonstrating multiple immunoregulatory mechanisms in action in our model. This finding is also consistent with long-term graft recipients showing systemic response to donor antigens, indicating the localized nature of graft protection, which is further consistent with our published studies with islets engineered to display on their surface SA-FasL or SA-PD-L1 (13, 25, 27, 29).

Collectively, these results demonstrate that transient co-display of SA-TM and SA-CD47 molecules on the islet surface operates in synergy to locally modulate both innate and adaptive immune responses that translate into sustained long-term graft survival. Inasmuch as the islet graft significantly contributes to immune rejection mechanisms by expressing various danger-associated molecular patterns, including HMGB1, tissue factor, and heat shock proteins (20, 22), engineering islets in a benign and practical fashion to co-display immune ligands with synergistic functions on their surface has significant clinical implications. This report is the first demonstration to our knowledge of using islets engineered with 2 immune ligands with distinct mechanisms of action as a platform to prevent graft rejection in the absence of immunosuppressive agents. The modular nature of ProtEx technology allows for displaying on the islet surface a collection of immune ligands with distinct mechanisms of action to achieve graft survival. This technology provides several advantages over ectopic expression of genes encoding immunomodulators, including (a) lack of genetic modification of islets that reduces the risk of unexpected negative consequences; (b) transient and positional display of immunomodulators to polarize immune response to a regulatory path without the risks associated with continuous expression, particularly in case of potent immunomodulators such as FasL; (c) precise control over the number of ligands to be displayed on islet surface; and (d) serving a simple, robust, and highly scalable platform, with the entire engineering procedure performed within 2 hours under extracorporeal conditions without a negative impact on the viability and metabolic function of islets. We routinely prepare batches of islets for multiple mouse transplants per batch without compromising islet health. In pilot studies, we also tested this process for engineering human, non-human primate (NHP), porcine, and rat islets at doses required to restore euglycemia in diabetic humanized mice, rats, and NHPs, without any procedural issues.

## Methods

### Sex as a biological variable.

Both male and female mice were used as islet donors in this study. Male mice were used as transplant recipients because intraportal transplantation requires delicate portal vein manipulation and male mice provide more consistent surgical access. Female mice can show greater procedural variability due to smaller vessel size and hormonal cycles. The study was not designed or powered to detect sex-based differences, and outcomes were therefore not analyzed separately by sex.

### Animals.

C57BL/6 (CD45.2), C57BL/6.SJL.FoxP3-GFP (CD45.1), and BALB/c mice were purchased from The Jackson Laboratory. Mice were housed and bred in a specific pathogen–free facility at the University of Missouri.

### Production and characterization of recombinant proteins.

Recombinant SA-TM and SA-CD47 proteins were produced in *Drosophila* S2 cells and purified using metal ion–charged Sepharose columns. Protein purity and structural integrity were assessed via SDS-PAGE and Western blot analyses, per published protocols (13).

### Islet isolation, engineering, and functional analysis.

Islets were isolated from BALB/c mice using Liberase TL (Roche, 1815032) using established protocols and kept overnight for recovery (38). For engineering, islets were first washed 3 times with sterile PBS by gravity sedimentation at room temperature (r.t.). Based on islet equivalent (IEQ) required for transplantation, appropriate IEQ were resuspended in 0.7–1 mL of freshly prepared 15 μM biotin in PBS, incubated for 30 minutes at r.t. on an angled tube rotator, and washed 3 times with PBS. Islets were then resuspended in PBS (~250 μL/500 islets) and transferred to FBS-precoated 14-mL round-bottom tubes followed by incubation with SA-TM (3.2 μg/500 IEQ), SA-CD47 (0.4 μg/500 IEQ), or SA-TM/SA-CD47 for 30 minutes at r.t. on a rotator. For combined engineering, islets were incubated with SA-TM for 5 minutes before adding SA-CD47. Control islets received an equimolar amount of unconjugated SA. An Alamar blue assay was used to assess metabolic activity using 50 islets per well in supplemented RPMI 1640 medium. Resorufin fluorescence (562 nm) was measured using a multi-mode reader (BioTek Synergy) after 6–8 hours. Glucose-stimulated insulin secretion assays were performed as published previously (13).

### Fluorescent immunostaining and confocal microscopy.

Details of all antibodies used in this study can be found in Supplemental Table 1. Engineered islets were incubated with SA–Texas red, anti–human CD47–APC, and anti–human TM–FITC. Antibody incubation was performed for 10 minutes at r.t. without agitation, followed by 15 minutes at 4°C on a rotator. Islets were then washed at least twice with 10 mL of calcium- and magnesium-free PBS and transferred to wells of a confocal microscopy plate. Confocal imaging was performed using a Leica SP8 or Zeiss LSM microscope equipped with appropriate laser lines for FITC, Texas red, and APC detection. Images were acquired using 40× or 63× oil immersion objectives. *Z*-stacks and multichannel images were captured where applicable and processed using ImageJ (NIH) or Zen (Zeiss) software.

### Islet transplantation.

C57BL/6 or C57BL/6.SJL.FoxP3-GFP mice were injected intravenously with STZ (200 mg/kg; Sigma-Aldrich) to induce diabetes. Mice with non-fasting blood glucose exceeding 300 mg/dL received intraportal transplants of 700 IEQ allogeneic BALB/c islets. Blood glucose monitoring was done biweekly for more than 300 days, with readings of greater than 250 mg/dL on 2 consecutive days indicating rejection.

### IPGTT.

Long-term graft recipients at the experimental endpoint were fasted for 6 hours and then subjected to an IPGTT, as previously published (28). Blood samples were obtained at 0, 10, 20, 30, 45, 60, 90, and 120 minutes after intraportal glucose injection (2 g/kg body weight). Blood glucose levels were measured using an Accu-Check Nano Glucometer and Smart View Test Strips (Roche Diagnostics).

### Quantitative RT-PCR.

Total RNA was extracted from the liver of mice at rejection or experimental endpoint using TRIzol reagent (Invitrogen) and RNeasy Mini Kit (Qiagen). The quantity of total RNA was assessed with a NanoDrop ND-2000c spectrophotometer (Thermo Fisher Scientific). Subsequently, 1 μg of RNA was converted into cDNA using SuperScript IV VILO cDNA Master Mix (Thermo Fisher Scientific). Quantitative RT-PCR was performed using a TaqMan assay (Thermo Fisher Scientific) to investigate the expression of various genes involved in pro- and antiinflammatory responses. Morpheus (https://software.broadinstitute.org/morpheus/) was used to generate the heatmap.

### Deep immunophenotyping.

Islet transplant recipients were euthanized on days 3, 12, at rejection, or experimental endpoint (>100 days) to collect the islet-graft-harboring liver and spleen. Liver was perfused with PBS and dispersed into single cells using 0.5 mg/mL collagenase IV (Sigma-Aldrich, C4-22-1G), per a previously described protocol (13). Single-cell suspensions prepared from the liver and the spleen were filtered through a 40-μm nylon mesh, and cells were collected by centrifugation (180*g* for 7 minutes). Splenocytes (1 × 10^6^) and liver-derived cells (2 × 10^6^) were then incubated first in Fc block (CD16/CD32, BD Bioscience) to minimize nonspecific binding, followed by fluorescently labeled antibodies against various cell surface markers. For intracellular staining, cells were subsequently fixed and permeabilized using Fixation/Permeabilization buffer (Thermo Fisher Scientific, 00-5523-00) for 30 minutes at 4°C, followed by washing with 1× permeabilization/wash buffer. Intracellular FoxP3 staining was performed by incubating cells with an anti-FoxP3 antibody (1 μL per sample) for 30 minutes at 4°C. Cells were then washed sequentially with 1× permeabilization buffer and 1× PBS, resuspended in PBS, and prepared for acquisition. Cells were acquired by Cytek Aurora flow cytometry and analyzed using FCS express (7). A comprehensive flow cytometric gating strategy for all analyzed cell populations is provided in Supplemental Figure 1.

### Statistics.

The normality of data was checked using the Kolmogorov-Smirnov test. An unpaired, 2-tailed Student’s *t* test was used for 2-group comparisons with normal distribution, while the Mann-Whitney *U* test was used for non-parametric data. One-way ANOVA was used to compare multiple groups, followed by Dunnett’s test for normally distributed data. Non-parametric data were analyzed using the Kruskal-Wallis test followed by Dunn’s test. The log-rank (Mantel-Cox) test was utilized to analyze graft survival between groups. Statistical analyses were conducted using GraphPad Prism version 9. All results are expressed as mean ± SD, with a *P* value of less than 0.05 considered statistically significant.

### Study approval.

All animal studies were reviewed and approved by the Institutional Animal Care and Use Committee (IACUC) at the University of Missouri, Columbia, Missouri, USA. Animal procedures were carried out in accordance with the NIH *Guide for the Care and Use of Laboratory Animals* (National Academies Press, 2011).

### Data availability.

All data supporting the findings of this study are included in the article and its supplemental materials. Source data for the figures and tables are provided in the Supporting Data Values file. Additional information related to this study is available from the corresponding author upon reasonable request.

## Author contributions

HS and ESY designed experimental methods. SK, MT, DB, VU, KMYF, AJN, and AT conducted the experiments, performed data analysis, and prepared figures. HS, ESY, SK, MT, and DB wrote the manuscript.

## Conflict of interest

The authors have declared that no conflict of interest exists.

## Funding support

This work is the result of NIH funding, in whole or in part, and is subject to the NIH Public Access Policy. Through acceptance of this federal funding, the NIH has been given a right to make the work publicly available in PubMed Central.

- NIH grant R01AI121281 (to HS).

## Supplementary Material

Supplemental data

Supporting data values

## Version 1 03/17/2026

In-Press Preview

## Version 2 05/08/2026

Electronic publication
