# Supplementary material for: Islets co-engineered with thrombomodulin and CD47 achieve sustained survival in allogeneic recipients without chronic immunosuppression
Source: JCI Insight. 2026 Mar 17;11(9):e200686. doi: 10.1172/jci.insight.200686 (PMC13232006; doi:10.1172/jci.insight.200686)
Supplement: Supplemental data [file jciinsight-11-200686-s097.pdf]

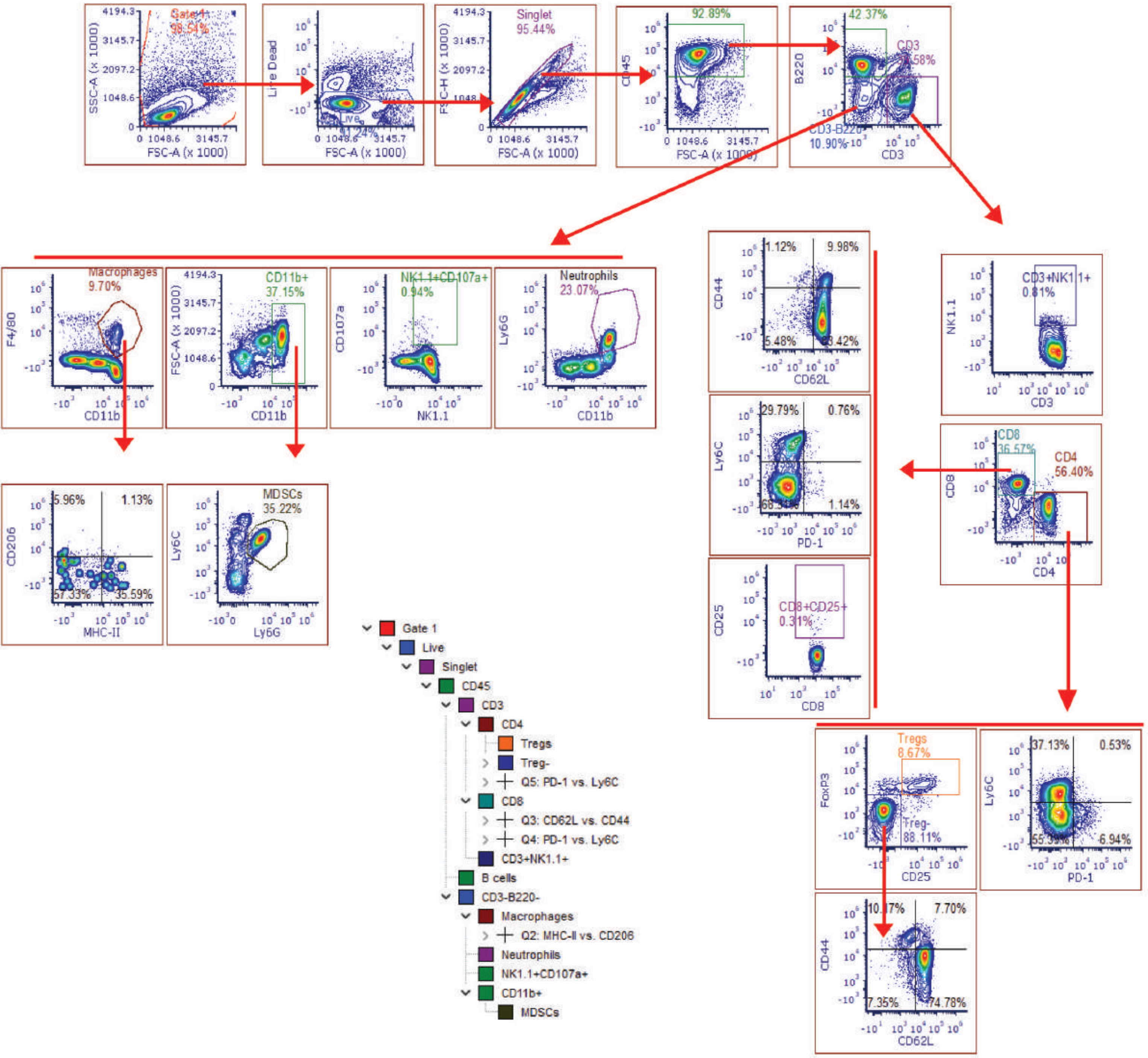


# Supplementary Figure S1

Flow cytometry gating strategy for immune cell profiling: Flow cytometry gating strategy for immune cell profiling. Single-cell suspensions were first gated on FSC-A vs SSC-A, followed by exclusion of dead cells using a viability dye. Live cells were gated for singlets (FSC-A vs FSC-H) and subsequently for CD45$^{+}$ leukocytes. From CD45$^{+}$cells, T cells were identified as CD3$^{+}$ and further subdivided into CD4$^{+}$ and CD8$^{+}$ T cells. CD4$^{+}$ T cells were analyzed for regulatory T cells defined as CD4$^{+}$CD25$^{+}$FoxP3$^{+}$, and expression of PD-1 and Ly6C where indicated. CD8$^{+}$ T cells were further characterized for activation/memory status using CD44 and CD62L, and PD-1 expression; a minor CD8+CD25+ population is also shown. NK cells were identified as CD3$^{+}$NK1.1$^{+}$ and assessed for degranulation by CD107a expression. B cells were defined as CD3-B220$^{+}$. Myeloid cells were gated from the CD3$^{-}$B220$^{-}$ fraction, with macrophages identified as CD11b$^{+}$F4/80$^{+}$and further characterized by MHC-II (M1) and CD206 (M2) expression. Neutrophils were defined as CD11b$^{+}$Ly6G$^{+}$, and myeloid-derived suppressor cells (MDSCs) as CD11b$^{+}$Ly6G$^{+}$Ly6C$^{+}$.

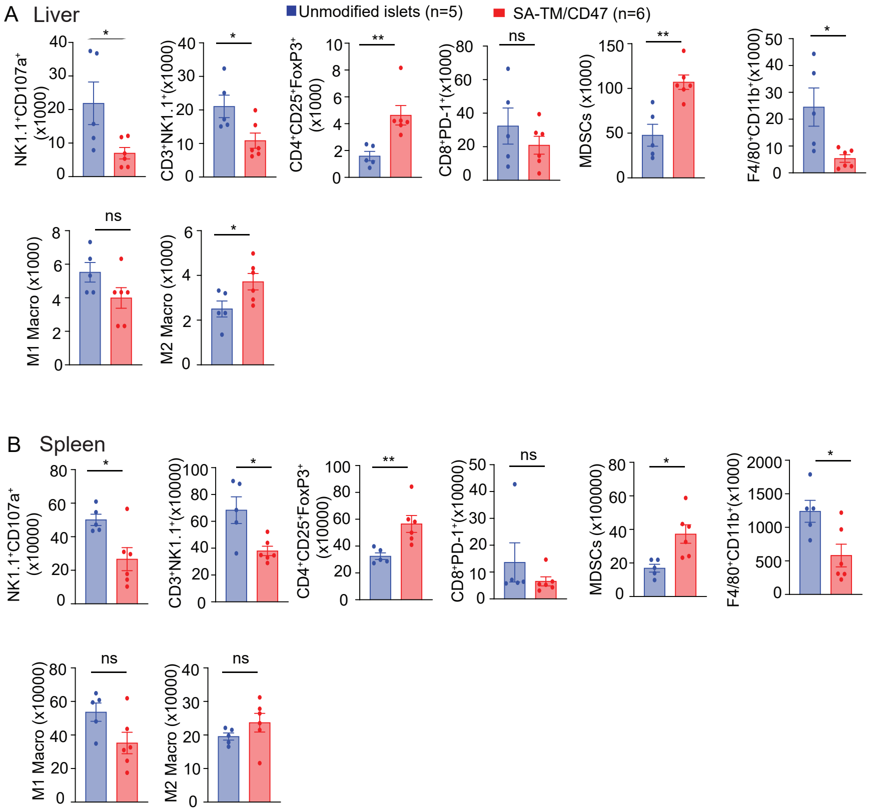


Supplementary Figure S2

Supplementary Figure S2: Deep immunophenotyping shows tolerogenic immune response in mice transplanted with SA-TM/CD47-engineered allogenic islets. Diabetic C57BL/6 mice received intraportal transplants of 700 IEQ of either allogeneic unmodified islets or islets co-engineered with SA-TM/CD47. At day 12 post-transplantation, immunophenotyping of graft-infiltrating and splenic immune cells demonstrated a predominantly tolerogenic immune response in the engineered islet group as absolute numbers . (A) Liver and (B) spleen immune cell composition was analyzed via flow cytometry. The data represent the absolute numbers of immune cells per tissue, shown as mean ± SD from n=4-5 graft recipients. Statistical significance was determined using a one-tailed unpaired Student’s t-test (*p < 0.05, **p < 0.01, ***p < 0.001, ****p < 0.0001).

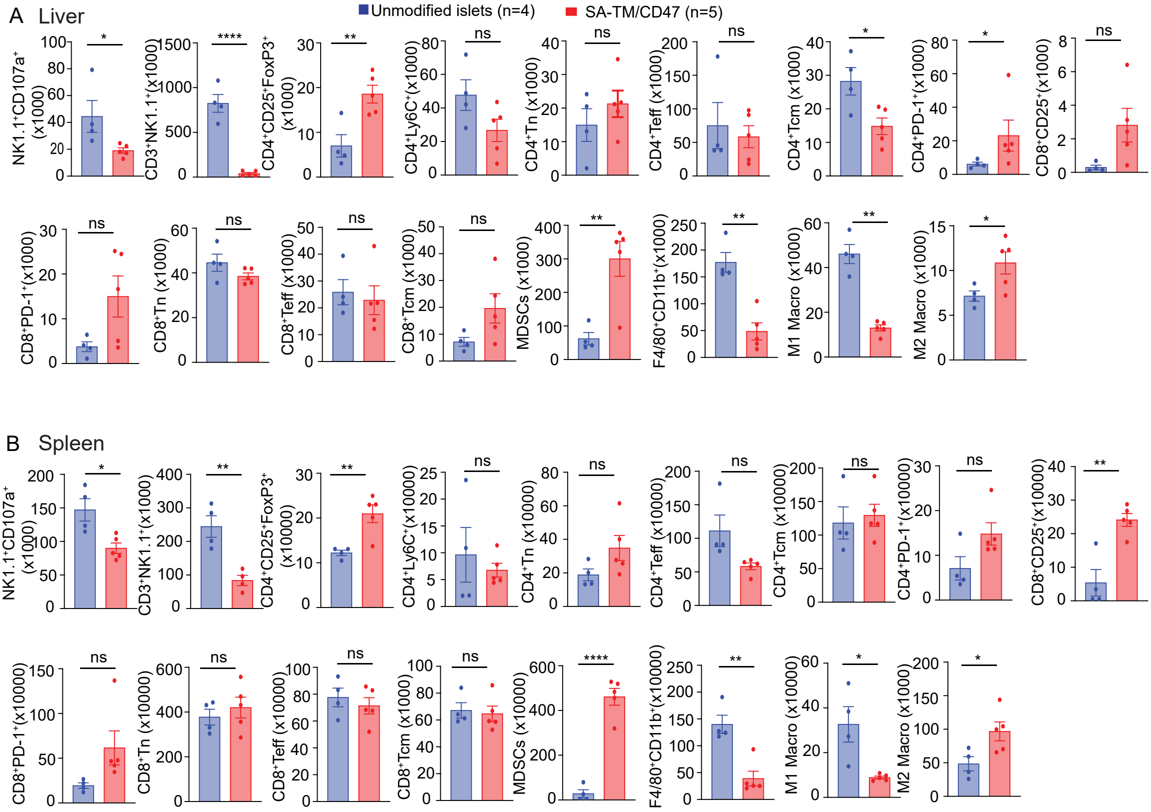


## Supplementary Figure S3

Supplementary Figure S3: Deep immunophenotyping shows tolerogenic immune response in mice transplanted with SA-TM/CD47-engineered allogenic islets. Diabetic C57BL/6 mice received intraportal transplants of 700 IEQ of either allogeneic unmodified islets or islets co-engineered with SA-TM/CD47. At day 12 post-transplantation, immunophenotyping of graft-infiltrating and splenic immune cells demonstrated a predominantly tolerogenic immune response in the engineered islet group as absolute numbers . (A) Liver and (B) spleen immune cell composition was analyzed via flow cytometry. The data represent the absolute numbers of immune cells per tissue, shown as mean ± SD from n=4-5 graft recipients. Statistical significance was determined using a one-tailed unpaired Student's t-test (*p < 0.05, **p < 0.01, ***p < 0.001, ****p < 0.0001).

| S.N. | Antibody | Host | Clone | Company | Catalog No. | Lot No. | Dilution |
|---|---|---|---|---|---|---|---|
| 1 | V500 Syrian Hamster anti mouse CD3e | Hamster | 500A2 | BD Horizon | 560771 | 8164558 | 1:50 |
| 2 | BUV496 Anti-mouse CD4 | Rat | GK1.5 | BD Biosciences | 612952 | 357490 | 1:200 |
| 3 | BUV615-Anti-mouse CD5 | Rat | 53-7.5 | BD Biosciences | 751298 | 1172962 | 1:200 |
| 4 | SparkViolet538 Anti-mouseCD8 | Rat | QA17A07 | BioLegend | 155019 | B329035 | 1:80 |
| 5 | PerCP5.5 Anti-mouse-CD11b | Rat | M1/70 | BD Pharmigen | 550993 | 6112802 | 1:100 |
| 6 | AF647 Anti-mouse CD11c | Hamster | N418 | BioLegend | 117312 | B273696 | 1:100 |
| 7 | BV570 Anti-mouse CD19 | Rat | 6D5 | BioLegend | 115535 | B315920 | 1:50 |
| 8 | PE/Cy5 Anti-mouse CD25 | Rat | PC61 | BioLegend | 102010 | B320333 | 1:200 |
| 9 | BB700 Anti-mouse CD44 | Rat | IM7 | BD Biosciences | 566506 | 1050286 | 1:60 |
| 10 | APC Fire810 Anti-mouse CD45 | Rat | 30-F11 | Biolegend | 103174 | B335319 | 1:50 |
| 11 | AF594 anti-mouse/human B220 | Rat | RA3-6B2 | BioLegend | 103254 | B327137 | 1:200 |
| 12 | BV711 Anti-mouse CD62L | Rat | MEL-14 | BioLegend | 104445 | B317393 | 1:400 |
| 13 | APC Fire750 Anti-mouse CD80 | Hamster | 16-10A1 | BioLegend | 104740 | B310036 | 1:50 |
| 14 | PerCP-eFluor 710-CD103 | Hamster | 2.00E+07 | Fischer Scientific | 46-1031-82 | 2332024 | 1:50 |
| 15 | PE/Cy7 Anti-mouse CD107a | Rat | 1D4B | BD Biosciences | 560647 | 314075 | 1:800 |
| 16 | BV785 Anti-mouse CD206 | Rat | C068C2 | BioLegend | 141729 | B329080 | 1:100 |
| 17 | APC Anti-mouse PD-1(CD279) | Rat | 29F.1A12 | BioLegend | 135210 | B307869 | 1:200 |
| 18 | AF700Anti-mouse NK1.1 | Mouse | PK136 | BioLegend | 108730 | B311346 | 1:800 |
| 19 | BV605 Anti-mouse Ly6C | Rat | HK1.4 | BioLegend | 128036 | B266313 | 1:20 |
| 20 | BUV395 Anti-mouse Ly6G | Rat | 1A8 | BD Biosciences | 563978 | 300815 | 1:100 |
| 21 | Pacific Blue Anti-mouse F4/80 | Rat | BM8 | BioLegend | 123124 | B280040 | 1:100 |
| 22 | Spark Blue 550 Anti-mouse I-A/I-E(MHC-II) | Rat | M5/114.15.2 | BioLegend | 107662 | B320821 | 1:50 |
| 23 | BUV563 Anti-mouse γδ T-Cell Receptor | Rat | V65 | BD Biosciences | 749464 | 1172963 | 1:20 |
| 24 | BUV661, Anti-mouse CCR7 | Rat | 4B12 | BD Biosciences | 741677 | 1214113 | 1:50 |
| 26 | Alexa Fluor488-anti-mouse/rat-FoxP3 | Rat | FJK-16s | eBioscience | 53-5773-82 | 2068006 | 1ul/$10^6$cells |
| 27 | BUV737 Rat Anti-Mouse CD335 (NKp46) | Rat | 29A1.4 | BD Biosciences | 612805 | 1118991 | 1:30 |

**Supplementary Table 1:** Antibodies used for flow cytometry. A comprehensive list of antibodies utilized for multi-color flow cytometry analyses in this study. The table provides details of antibody specificity, host species, clone designation, supplier, catalog and lot numbers, and working dilutions.
